# LINC01393, a Novel Long Non-Coding RNA, Promotes the Cell Proliferation, Migration and Invasion through MiR-128-3p/NUSAP1 Axis in Glioblastoma

**DOI:** 10.3390/ijms24065878

**Published:** 2023-03-20

**Authors:** Deheng Li, Junda Hu, Sen Li, Changshuai Zhou, Mingtao Feng, Liangdong Li, Yang Gao, Xin Chen, Xiaojun Wu, Yiqun Cao, Bin Hao, Lei Chen

**Affiliations:** 1 Department of Neurosurgery, Fudan University Shanghai Cancer Center, Shanghai 200032, China; 2 Department of Oncology, Shanghai Medical College of Fudan University, Shanghai 200032, China

**Keywords:** competing endogenous RNA, glioblastoma, LINC01393, miR-128-3p, nucleolar and spindle-associated protein 1

## Abstract

Nucleolar and spindle-associated protein 1 (NUSAP1) is a potential molecular marker and intervention target for glioblastoma (GBM). In this study, we aim to investigate upstream regulatory lncRNAs and miRNAs of NUSAP1 through both experimental and bioinformatic methods. We screened upstream lncRNAs and miRNAs of NUSAP1 through multiple databases based on ceRNA theory. Then, in vitro and in vivo experiments were performed to elucidate the relevant biological significance and regulatory mechanism among them. Finally, the potential downstream mechanism was discussed. LINC01393 and miR-128-3p were screened as upstream regulatory molecules of NUSAP1 by TCGA and ENCORI databases. The negative correlations among them were confirmed in clinical specimens. Biochemical studies revealed that overexpression or knockdown of LINC01393 respectively enhanced or inhibited malignant phenotype of GBM cells. MiR-128-3p inhibitor reversed LINC01393 knockdown-mediated impacts on GBM cells. Then, dual-luciferase reporter assay and RNA immunoprecipitation assay were conducted to validate LINC01393/miR-128-3p/NUSAP1 interactions. In vivo, LINC01393-knockdown decreased tumor growth and improved mice survival, while restoration of NUSAP1 partially reversed these effects. Additionally, enrichment analysis and western blot revealed that the roles of LINC01393 and NUSAP1 in GBM progression were associated with NF-κB activation. Our findings showed that LINC01393 sponged miR-128-3p to upregulate NUSAP1, thereby promoting GBM development and progression via activating NF-κB pathway. This work deepens understanding of GBM mechanisms and provides potential novel therapeutic targets for GBM.

## 1. Introduction

Glioblastoma (GBM) is the most common primary malignant brain tumor with high rates of recurrence and mortality [1]. Patients with GBM only have an overall median survival of less than 2 years despite proactive multimodal therapies, including surgical resection, chemotherapy, radiotherapy, targeted therapy, and tumor treating field (TTF) [2,3,4]. Therefore, exploring the molecular mechanism in-depth that contributes to GBM development and progression may provide new insights into therapeutic strategies.

Nucleolar and spindle-associated protein 1 (NUSAP1) is a 55-kD microtubule-associated protein (MAP), which is involved in mitotic progression, the formation and stability of spindle organization [5]. In recent years, NUSAP1 has been considered as an important regulator in the occurrence and development of malignant tumors. Several studies have found that NUSAP1 is highly expressed in liver cancer [6], cervical cancer [7], breast cancer [8], and melanoma [9], which is closely related to tumor progression and metastasis [7]. Also, NUSAP1 plays a role as a tumor antigen in acute myeloid leukemia (AML), and studies have confirmed that the production of its specific antibody is related to the sustained remission of the disease, and the detection of NUSAP1 antibody is beneficial to monitor the progression of AML [10].

The expression of NUSAP1 is significantly increased in GBM [11,12], which indicates that NUSAP1 could serve as a potential molecular marker and intervention target for GBM. Elevated expression of NUSAP1 was reported to increase proliferation, migration, and invasion of GBM cells [11,12]. A possible mechanism for NUSAP1’s carcinogenic role is activation of the hedgehog signaling pathway [13]. Zhao et al. reported that NUSAP1 potentiated chemotherapeutic resistance to temozolomide (TMZ) and doxorubicin (DOX) through its SAP domain [11]. However, the potentially upstream regulatory mechanism of NUSAP1 expression remains unclear. 

Long non-coding RNAs (lncRNAs) are a class of recently identified RNA molecules with a molecular weight of over 200 nucleotides in length, with no or limited protein-coding capacity [14,15]. While a proportion of lncRNA transcripts have been functionally characterized, the role of a large number of lncRNAs in cancers is unclear to date. For a dozen years, evidence has been revealed that lncRNAs can function as miRNA sponges and inhibit the binding of miRNAs to their target mRNAs [16]. Increasing evidence suggest the important role of lncRNA/miRNA/target gene axis in the progression of GBM [17]. For example, the lncRNA OXCT1-AS1/miR-195/CDC25A axis contributes to tumorigenesis in GBM [18] and lncRNA MALAT1 acts as a competitive sponge for miR-199a, and promotes proliferation and invasion of GBM cells by enhancing ZHX1 expression [19]. 

Studies have confirmed that multiple miRNAs can target NUSAP1 acting as an upstream regulators in various malignant tumors except glioma, such as miR-193a-5p [6], miR-758-3p [20], miR-569 [21], and miR-769-5p [22]. Here, we conducted bioinformatic analysis of *TCGA LGG-GBM* cohort and *ENCORI* database and screened that a novel lncRNA LINC01393 which may serve as a sponge for miR-128-3p in the regulation of NUSAP1 expression. Further, we demonstrated in vitro and in vivo that LINC01393 indeed regulates NUSAP1 expression by acting as a competing endogenous RNA (ceRNA) against miR-128-3p, thereby activating the NF-κB pathway to promote the development and progression of GBM. Our study reveals the biological function and potential regulatory mechanism of LINC01393 in glioma for the first time, which may provide a novel diagnostic biomarker and potential therapeutic target for glioma.

## 2. Results

### 2.1. NUSAP1 Expression Is Elevated in Glioma with Higher Malignancy

**Figure 1:**
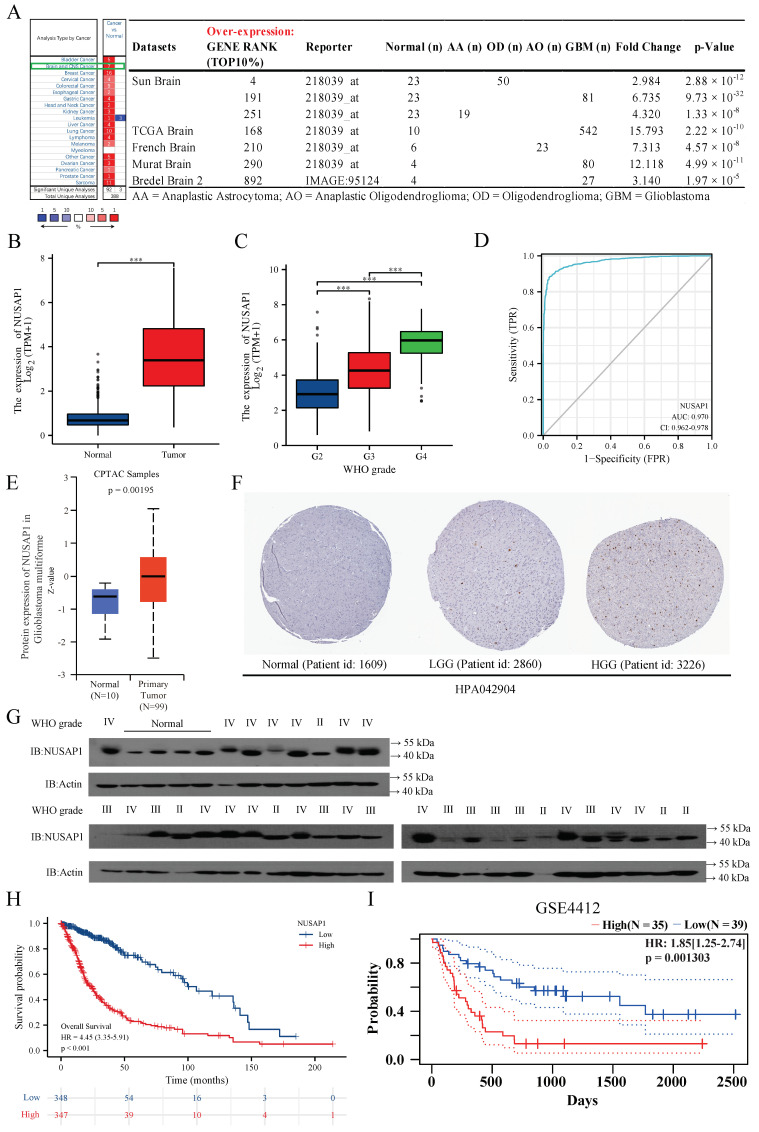
High expression of NUSAP1 correlates with higher tumor grade and poor prognosis of glioma. (**A**) The transcription levels of NUSAP1 in 20 different types of cancers and 7 datasets of glioma of Oncomine. (**B**) Comparison of NUSAP1 expression in GBM and normal tissues from TCGA+GTEx database. (**C**) Expression of NUSAP1 in different grade gliomas from TCGA database. (**D**) ROC curve is applied to evaluate the diagnostic value of NUSAP1 for glioma. (**E**) Comparison of NUSAP1 protein level in GBM and normal tissues of CPTAC datasets with the online tool of UALCAN. (**F**) Protein levels of NUSAP1 in normal tissues, LGG and HGG from the HPA database. (**G**) Western blot analysis of NUSAP1 protein lysates from glioma tissues and normal brain tissues. (**H**) Kaplan-Meier survival analysis of overall survival duration in TCGA glioma patients according to NUSAP1 expression. (**I**) The relationship between NUSAP1 expression and prognosis of glioma in PrognoScan is validated by GSE4412. *** *p* < 0.001.

NUSAP1 mRNA expression was significantly higher in multiple glioma types (AA, AO, OD, and GBM) than normal brain tissue in seven glioma datasets of *Oncomine* (Figure 1A) as well as in the merged *GTEx* and *TCGA* LGG-GBM datasets (Figure 1B). In addition, data from the *TCGA* also showed NUSAP1 mRNA expression levels significantly increased with higher WHO grade of glioma (Figure 1C). For NUSAP1 potential diagnostic value, ROC analysis revealed an area under curve of 0.970 (95% CI 0.962–0.978), with sensitivity and specificity of 0.884 and 0.962, respectively (Figure 1D). Consistent with overexpression at the RNA level, NUSAP1 protein in GBM tissues was confirmed in CPTAC datasets with the online tool of UALCAN (Figure 1E). These findings were also observed by immunohistochemistry staining data from *HPA* database (Figure 1F) and western blot analysis (WHO II, n = 6; WHO III, n = 9; WHO IV, n = 17; normal brain tissues, n = 4; Figure 1G). Moreover, higher NUSAP1 expression indicated worse prognosis of glioma from *TCGA* (HR = 4.45, 95% CI = 3.35 to 5.91, *p* < 0.001; Figure 1H), and its prognostic role was validated by GSE4412 using PrognoScan (*p* = 0.001303; Figure 1I). Altogether, these results suggest that a high level of NUSAP1 is critically involved in GBM progression.

### 2.2. LINC01393 May Regulate the Expression of NUSAP1 by Acting as a ceRNA of miR-128-3p in Glioma

**Figure 2:**
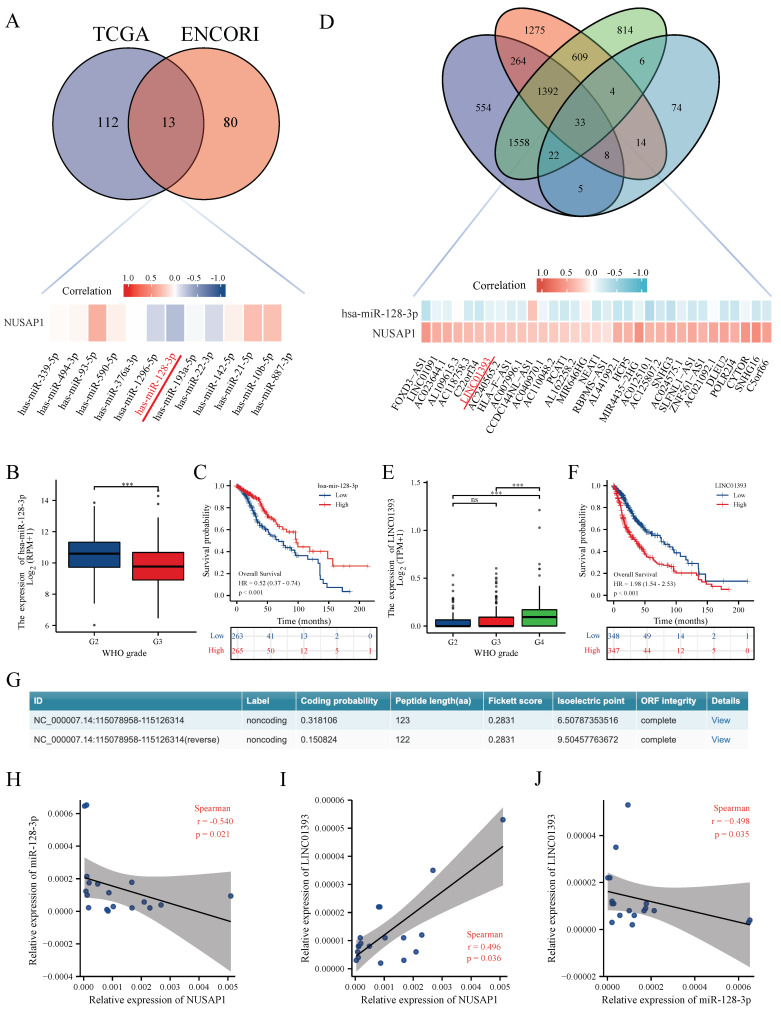
LINC01393 may regulate the expression of NUSAP1 by acting as a ceRNA of miR-128-3p in glioma. (**A**) Thirteen candidate miRNAs are screened by TCGA LGG-GBM cohort and ENCORI database, and correlation between candidate miRNAs and NUSAP1 is displayed as heatmap. (**B**,**C**) Downregulated miR-128-3p correlated with higher tumor grade and poor prognosis of glioma from TCGA. (**D**) Thirty-three candidate lncRNAs are screened by TCGA LGG-GBM cohort and ENCORI database, and correlation among miR-128-3p, NUSAP1, and candidate lncRNAs are displayed as heatmap. (**E**,**F**) High expression of LINC01393 correlated with higher tumor grade (**E**) and poor prognosis (**F**) of glioma from TCGA. (**G**) Protein coding ability prediction of LINC01393 by CPC 2.0. (**H**–**J**) qPCR correlation analysis among LINC01393, miR-128-3p, and NUSAP1 in human GBM samples. *** *p* < 0.001, ns: *p* > 0.005.

To explore the upstream miRNAs of NUSAP1, we used *ENCORI* and *TCGA* (see Method: 2) to predict the potential miRNAs interacting with NUSAP1, and 13 candidate miRNAs was illustrated in Figure 2A. miR-128-3p, which has highest negative correlation coefficient with NUSAP1 was selected for further investigation. To improve visualization, a miRNA-NUSAP1 correlation heat map was established using R software. In addition, the expression and prognostic value of miR-128-3p in glioma were determined in TCGA. As presented in Figure 2B,C, miR-128-3p was markedly downregulated in glioma and its downregulation was positively linked to patients’ better prognosis.

Next, the upstream lncRNAs of NUSAP1 and miR-128-3p were also predicted using *ENCORI* and *TCGA* based on the criteria as mentioned in method section (see Method: 2). Thirty-three predictive lncRNAs were achieved, and LINC01393, which has positive correlation with NUSAP1 and negative relationship with miR-128-3p, was selected as our research target. A lncRNAs-miR-128-3p-NUSAP1 correlation heat map was established using R software as well (Figure 2D). LINC01393 was markedly upregulated in glioma and its overexpressing was negatively linked to patients’ better prognosis (Figure 2E,F). The Cox regression showed LINC01393 was an independent prognostic factor in patients with glioma (HR = 1.795, 95%CI = 1.139–2.717, *p* = 0.011) (Table 1). Bioinformatics analysis using *CPC 2.0* and *InterPro* indicated that LINC01393 lacks ability to encode protein (Figure 2G and S1). To verify these predictions, qRT-PCR was also performed in 18 GBM samples to verify the correlation between LINC01393, miR-128-3p and NUSAP1 (Figure 2H–J). All these findings suggest that LINC01393 might be served as a miR-128-3p sponge to promote the progression of glioma via upregulating NUSAP1.

### 2.3. LINC01393, miR-128-3p, and NUSAP1 Expression in Glioma Cells

**Figure 3:**
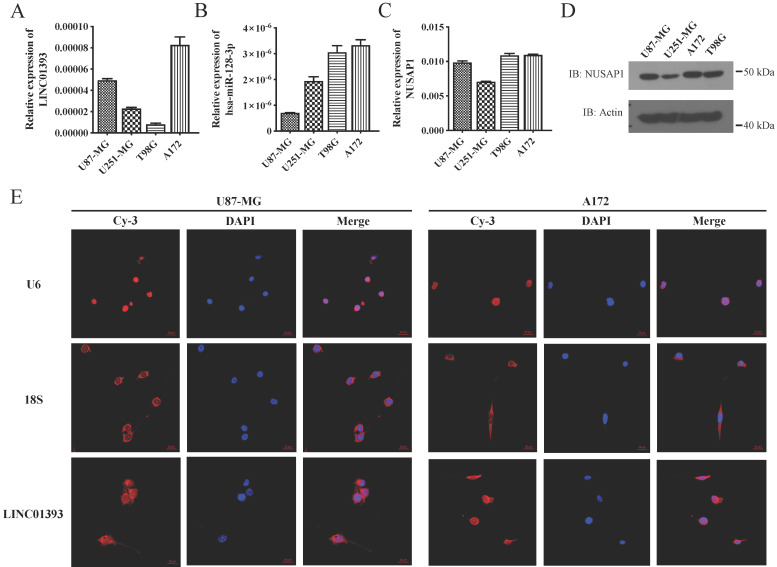
Expression of LINC01393, miR-128-3p and NUSAP1 in different glioma cell lines. (**A**–**C**) qRT-PCR analysis of LINC01393 (**A**), miR-128-3p (**B**) and NUSAP1 (**C**) expression levels in U87-MG, U251-MG, T98G and A172 glioma cells. (**D**) Western blot analysis of NUSAP1 protein expression in glioma cells. (**E**) RNA fluorescence in situ hybridization (FISH) images show that LINC01393 in U87-MG and A172 cells express in the cytoplasm. U6 and 18s rRNA are set as reference genes of the nucleus and cytoplasm respectively. Nuclei are stained with DAPI. Actin is used as a loading control.

Four different glioma cell lines were utilized to address the assumed roles of LINC01393, miR-128-3p and NUSAP1 signaling in the regulation of GBM cells progression. qRT-PCR were applied to detect expression levels of LINC01393, miR-128-3p and NUSAP1 mRNA in these GBM cell lines. The abundance of LINC01393 were relatively lower in U251-MG and T98G, but higher in other two cell lines (Figure 3A). All the four cell lines harbor expression of miR-128-3p with varied amount from each other (Figure 3B). NUSAP1 expression was detected in both mRNA and protein levels, which showed similar quantity in U87-MG, T98G and A172 cell lines, but higher than the U251-MG cell line (Figure 3C,D). To show the localization of LINC01393, the probes were applied to fixed U87-MG and A172 cells. The immunofluorescent images showed significant LINC01393 signals with main distribution in the cytoplasm (Figure 3E). These results demonstrated that GBM cells express LINC01393, miR-128-3p and NUSAP1 under normal condition.

### 2.4. LINC01393 Promoted Proliferation, Migration and Invasion of GBM Cells

**Figure 4:**
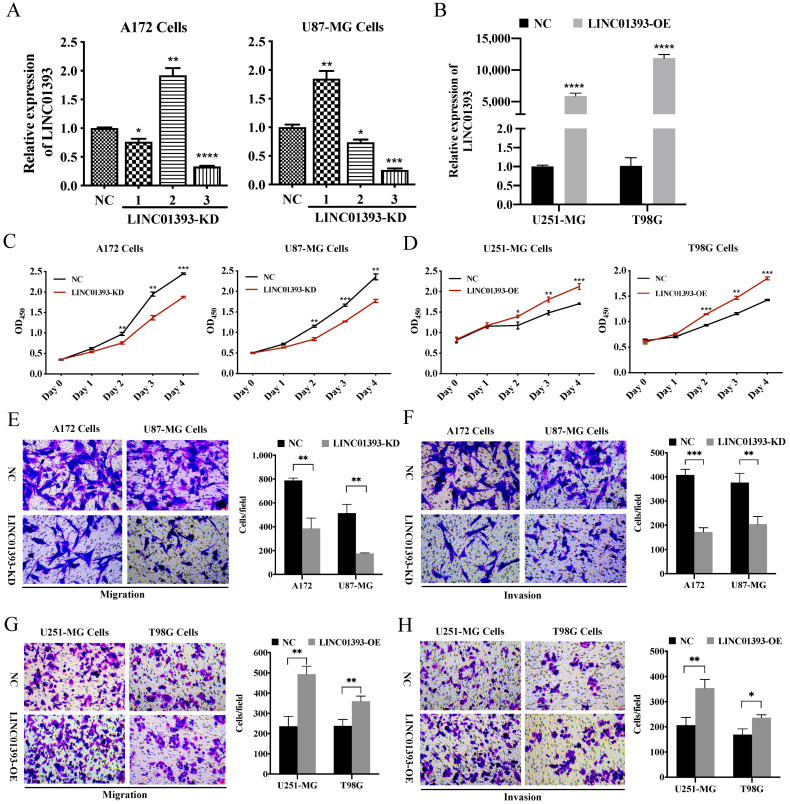
Effects of LINC01393 on proliferation, migration, and invasion of GBM in vitro. (**A**,**B**) Transfection efficiency of LINC01393 is analyzed using qRT-PCR in GBM cells. A172 and U87-MG cells are selected for LINC01393-KD, and U251-MG and T98G are selected for LINC01393-OE. (**C**,**D**) CCK-8 assays to assess the proliferation rate of LINC01393-KD (**C**) or OE (**D**). (**E**–**H**) Transwell migration and Matrigel invasion assays are used to measure the migration and invasion ability of GBM cells with LINC01393-KD (**E**,**F**) and LINC01393-OE (**G**,**H**). Representative staining images are presented (Scale bar = 50 μm). All data are represented as mean ± SEM of 3 independent experiments. * *p* < 0.05; ** *p* < 0.01; *** *p* < 0.001; **** *p* < 0.0001, relative to control.

A172 and U87-MG cells with high level of LINC01393 expression were selected for LINC01393-KD, while U251-MG and T98G were selected for LINC01393-OE. To explore the biological functions of the newly discovered LINC01393, efficacy of LINC01393 knockdown in A172 and U87-MG cells by shRNAs as determined by qRT-PCR. LINC01393-KD3 exhibiting the strongest knockdown efficacy was used for subsequent experiments (Figure 4A). Furthermore, we successfully constructed a LINC01393 expression lentivirus and overexpressed it in U251-MG and T98G cells. (Figure 4B). Cell Counting Kit8 (CCK-8) assay showed knockdown of LINC01393 markedly inhibited A172 and U87-MG proliferation; conversely, LINC01393 overexpression promoted cell proliferation (Figure 4C,D).

To examine LINC01393’s potential role in GBM cells migration and invasion, we conducted transwell cell migration and Matrigel-coated invasion assays and found that LINC01393-KD dramatically attenuated the tumor cell migration and invasion ability compared with NC (Figure 4E,F). Moreover, LINC01393-OE markedly accelerated the migration and invasion abilities of U251-MG and T98G cells (Figure 4G,H). Collectively, these results indicate that LINC01393 plays a vital role in regulating proliferation, migration and invasion of GBM cells.

### 2.5. LINC01393 Functions as a ceRNA against miR-128-3p to Promote NUSAP1 Expression In Vivo

**Figure 5:**
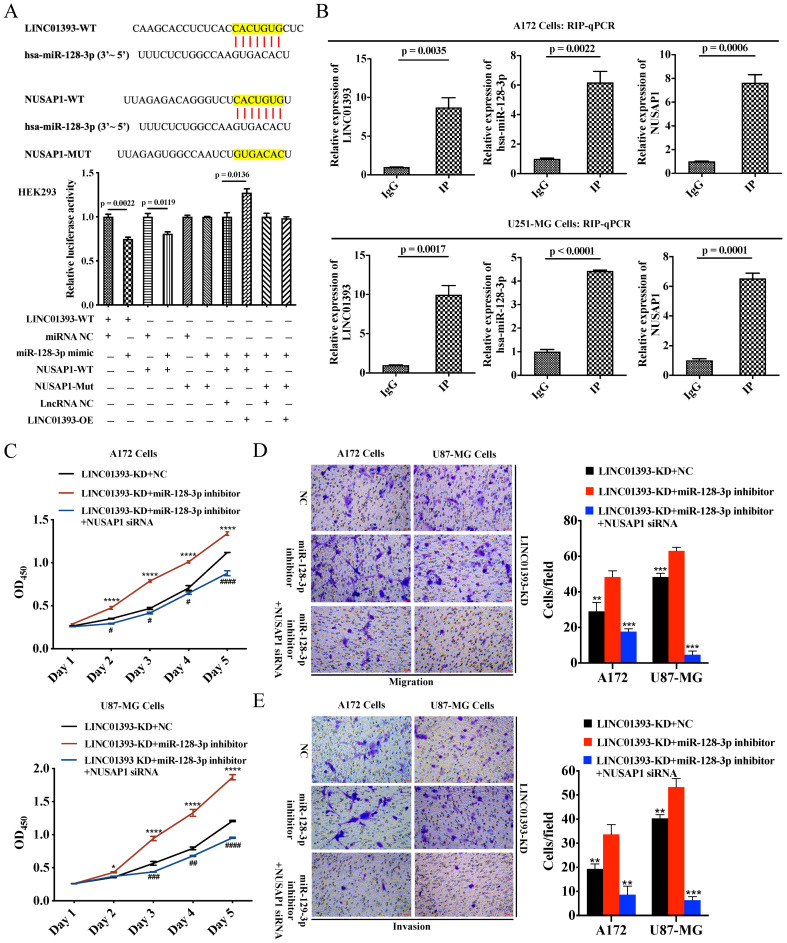
LINC01393 functions as a ceRNA against miR-128-3p to promote NUSAP1 expression in vitro. (**A**) Results of the dual-luciferase reporter gene assay in HEK-293 cells. (**B**) RIP-qPCR assay for LINC01393, miR-128-3p and NUSAP1 expression using anti-Ago2, compared to anti-IgG in cells. (**C**) CCK-8 assays to assess the proliferation rate of LINC01393-KD cells (A172, U87-MG) after transfected with the indicated empty, miR-128-3p inhibitor, miR-128-3p inhibitor+NUSAP1-siRNA. (**D**,**E**) Transwell migration. (**D**) and Matrigel invasion assays. (**E**) of LINC01393-KD cells (A172, U87-MG) after transfected with the indicated empty, miR-128-3p inhibitor, miR-128-3p inhibitor+NUSAP1-siRNA. Representative staining images are presented (Scale bar = 50 μm). All data are represented as mean ± SEM of 3 independent experiments. * *p* < 0.05; ** *p* < 0.01; *** *p* < 0.001; **** *p* < 0.0001, # *p* < 0.05, ## *p* < 0.01, ### *p* < 0.001, #### *p* < 0.0001, relative to control.

According to the bioinformatics predictive analysis mentioned above, LINC01393 might be served as a miR-128-3p sponge to promote the progression of glioma via upregulating NUSAP1. We conducted luciferase reporter assays to verify the binding of LINC01393, miR-128-3p and NUSAP1 in HEK293 cells. Assay results revealed that miR-128-3p mimics significantly reduced the luciferase activity of LINC01393-WT and NUSAP1-WT, but not of NUSAP1-Mut; LINC01393-OE increased the luciferase activity of NUSAP1-WT/miR-128-3p mimic, but not of NUSAP1-Mut/miR-128-3p mimic (Figure 5A). Furthermore, we verified the direct interaction among LINC01393, hsa-miR-128-3p and NUSAP1 in GBM cells. The RNA-binding protein immunoprecipitation (RIP) assay was used to determine whether LINC01393, miR-128-3p and NUSAP1 participate in RNA-induced silencing complex (RISC) formation. The results showed an enrichment of LINC01393, miR-128-3p and NUSAP1 with anti-Ago2 compared with the anti-immunoglobulin G (IgG) control (Figure 5B).

In vitro experiments, we conducted to investigate the biological impact of LINC01393/miR-128-3p/NUSAP1 axis on GBM cells. We performed rescue experiments by miR-128-3p inhibitor administration alone or its combination with NUSAP1-siRNA in LINC01393-KD A172 and U87-MG cells. The application of miR-128-3p inhibitor remarkably enhanced proliferation, migration, and invasion of LINC01393-KD A172 and U87-MG cells. These effects were reversed by the concomitant inhibition of NUSAP1 (Figure 5C–E). These results provide strong evidence suggesting that LINC01393 promotes NUSAP1 expression by inhibiting miR-128-3p and LINC01393/miR-128-3p/NUSAP1 axis may significantly contribute to progression of GBM.

### 2.6. Knockdown of LINC01393 Inhibits GBM Progression In Vivo, and could Be Partly Reversed by NUSAP1 Overexpression

**Figure 6:**
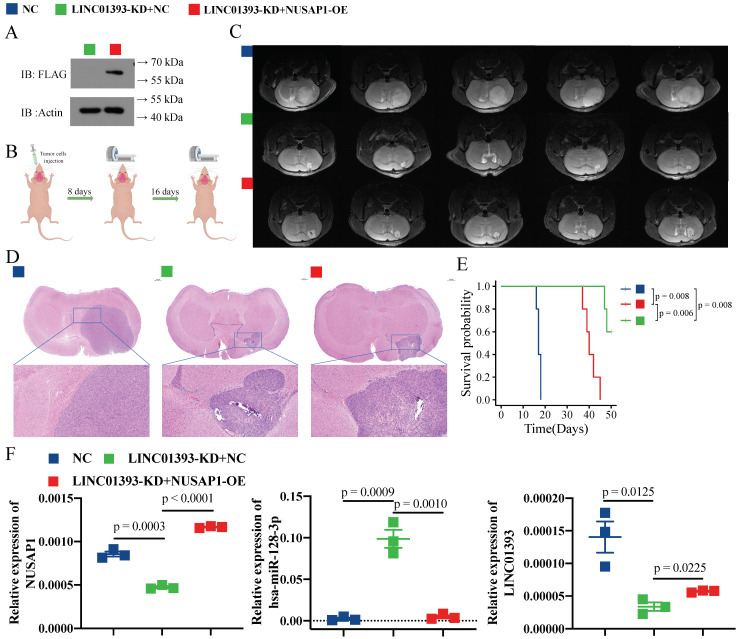
Knockdown of LINC01393 inhibits GBM Progression in vivo. (**A**) Western blot confirmation of NUSAP1 protein expression in U87-LINC01393-KD+NC and U87-LINC01393-KD+NUSAP1-OE cells. (**B**) Schematic view of orthotopic intracranial glioma model. (**C**,**D**) Representative axial T2-weighted MR images at 16 days (**C**) and hematoxylin and eosin (H&E) staining (**D**) of xenograft GBM tumors in normal control, LINC01393-KD+NC, and LINC01393-KD+NUSAP1-OE groups. (**E**) Survival curves of mice bearing normal control, LINC01393-KD+NC, and LINC01393-KD+NUSAP1-OE groups. (**F**) qRT-PCR analysis of LINC01393, miR-128-3p, and NUSAP1 levels in tumor tissues of nude mice injected with normal control, LINC01393-KD+NC, and LINC01393-KD+NUSAP1-OE.

After transfected with Lenti-sh-LINC01393-NC or/and Lenti-sh-LINC01393-NUSAP1-OE, NUSAP1 protein expression in U87-MG cells was confirmed by western blot (Figure 6A). LINC01393-KD+NC or LINC01393-KD+NUSAP1-OE cells were injected into the left striatum of mice, and tumors were monitored at 8 days (Figure S2) and 16 days via MRI (Figure 6B). Brain MRI at 16 days and H&E staining of the tumors showed that tumor volume in the LINC01393-KD+NC group was significantly smaller than that in the NC group and the LINC01393-KD+NUSAP1-OE group (Figure 6C,D). There was also a higher survival time in the LINC01393-KD group than in the LINC01393-KD+NUSAP1-OE group and the NC group (Figure 6E). Further, the expression level of LINC01393, miR-128-3p, and NUSAP1 of orthotopically transplanted tumor were tested by qRT-PCR (Figure 6F). As expected, NUSAP1 expression was significantly lower in the LINC01393-KD+NC group compared to the LINC01393-KD+NUSAP1-OE group and the NC group. MiR-128-3p expression showed a completely opposite trend. Overall, these findings indicate that blocking LINC01393/miR-128-3p/NUSAP1 axis can inhibit GBM development and progression.

### 2.7. LINC01393/miR-128-3p/NUSAP1 Axis induce NF-κB Pathway Activation

**Figure 7:**
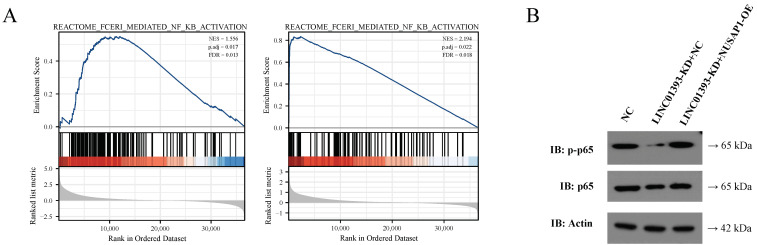
LINC01393 and NUSAP1 promotes NF-κB pathway. (**A**) GSEA of LINC01393 and NUSAP1 reveal an enrichment of NF-κB pathway. (**B**) Western blot of phospho-NF-κB-p65 and NF-κB-p65 in U87 cells following transfection with normal control, LINC01393-KD+NC, and LINC01393-KD+NUSAP1-OE.

GSEA based on NUSAP1 and LINC01393 expression in the TCGA LGG-GBM database was performed to explore the potential molecular pathways in gliomas. Interestingly, upregulated NUSAP1 and LINC01393 were both associated with NF-κB signaling pathway (Figure 7A). The expressions of p65 and phosphorylated p65 in NF-κB signaling pathway were further detected by immunoblotting. The result revealed that phosphorylated p65 expression decreased in LINC01393-KD+NC cells, while NUSAP1 overexpression could restore the phosphorylation of p65 (Figure 7B). These findings demonstrate that LINC01393/miR-128-3p/NUSAP1 axis induce the NF-κB pathway activation.

## 3. Methods

### 3.1. NUSAP1 Expression of Multiple Databases Analysis

The expression of NUSAP1 in 5 glioma datasets from *Oncomine* was analyzed online. The NUSAP1 RNA-seq data of gliomas in the *TCGA* LGG-GBM cohort and normal brain tissues in GTEx dataset were achieved from *UCSC XENA* (https://xenabrowser.net/datapages/ (accessed on 19 June 2021)). The proteomic data for NUSAP1 in both normal brain and GBM tissues were downloaded from *CPTAC* Data Portal (http://ualcan.path.uab.edu/analysis-prot.html (accessed on 31 December 2021)). In addition, we also validated the protein expression of NUSAP1 with immunohistochemistry (IHC) from The Human Protein Atlas (HPA) database (https://www.proteinatlas.org/ (accessed on 15 March 2022)). Kaplan-Meier plots were used to assess the overall survival (OS) for glioma patients in *TCGA*. The R ‘survminer’ and ‘survival’ packages were used for visualization and statistical analysis. GSE4412 was used to verify the correlation between NUSAP1 expression and OS through *PrognoScan* website (http://dna00.bio.kyutech.ac.jp/PrognoScan/index.html (accessed on 14 March 2022)).

### 3.2. Candidate Upstream miRNAs and lncRNAs Prediction of NUSAP1

*ENCORI* (http://starbase.sysu.edu.cn/) is an open-source platform for studying the miRNA-target interactions. Upstream potential binding miRNAs of NUSAP1 were predicted by *ENCORI* and *TCGA* LGG-GBM cohort database. First, we obtained NUSAP1-miRNA targets by intersecting *ENCORI*-predicted miRNAs with the prognostic significance of miRNAs in the *TCGA* LGG-GBM cohort. miRNAs with a significant negative strong correlation were selected. miRNA target of lncRNAs were also predicted by *ENCORI* and *TCGA* LGG-GBM cohort. The candidate lncRNAs were screened as follows: (1) positive correlation of lncRNAs with NUSAP1 expression in *TCGA* LGG-GBM cohort; (2) prognostic significance of lncRNAs in *TCGA* LGG-GBM cohort; (3) differential lncRNAs expression between WHO II and III, IV grade; (4) predicted binding lncRNAs of selected miRNA by *ENCORI*. Then, the Coding Potential Calculator [23] (*CPC*, version 2.0, http://cpc.cbi.pku.edu.cn/ (accessed on 9 October 2022)) and *Interpro* [24] (https://www.ebi.ac.uk/interpro/ (accessed on 9 October 2022)), were used to evaluate the coding potential of the predicted lncRNA.

### 3.3. Human Tissues Collection

Tissue specimens were collected with the informed consent of patients according to the Ethics Committee at Shanghai Cancer Center (ethic numbers: 050432-4-2108 *). A total of 33 glioma tissues (6 WHO grade II, 9 WHO grade III and 18 GBM) and 4 normal brain tissues were obtained from Department of Biobank, Fudan University Shanghai Cancer Center between 2017 and 2022. As soon as the surgical specimens were removed, they were immediately placed in liquid nitrogen. The glioma specimens were histologically classified and graded according to WHO classification guidelines by 2 experienced clinical pathologists blindly. Detailed patient information is provided in Table S1.

### 3.4. Cell Culture

The Cell Bank of the Shanghai Branch of the Chinese Academy of Sciences provided four GBM cell lines: U87-MG, T98G, U251-MG and A172. All cell lines tested negative for mycoplasma. Dulbecco’s modified Eagle’s medium (DMEM) was used to maintain cells, and was complemented with 10% FBS (Invitrogen, Carlsbad, CA, USA) and 1% Penicillin-Streptomycin. All cells were cultured at 37 °C in a humidified chamber with 5% CO_2_.

### 3.5. Fluorescence In Situ Hybridization (FISH)

The FISH assay was performed in U87-MG and A172 cells based on the manufacturer’s instructions to detect the subcellular localization of LINC01393. The LINC01393 probe (GUGAGUCGUGUCUCUCUUUUCUC) used in this study were synthesized and labeled with Cy3 (RiboBio, Guangzhou, China). Briefly, the cells were fixed with freshly prepared 4% paraformaldehyde for 15 min. Following prehybridization in PBS, hybridization solution was added to the cells and incubated at 37 °C for 30 min. Then, the cells were incubated with the Cy3-labelled probe in hybridization solution at 37 °C overnight. The next day, the cell nuclei were stained with DAPI (4′,6-diamidino-2-phenylindole) (Beyotime, Nantong, China) and observed under a fluorescence microscope (Leica, Wetzlar, Germany).

### 3.6. RNA Extraction and Quantitative Real-Time Polymerase Chain Reaction (qRT-PCR) Assays

Total RNA was isolated from human glioma cells using TRIzol reagent (Invitrogen, Carlsbad, CA, USA) following the manufacturer’s protocol. UV spectrophotometry was used to measure RNA concentration and quality. cDNA was generated from 1 μg RNA and qRT-PCR was performed on the 7500 Fast Real-Time PCR system (Applied Biosystems, Foster City, CA, USA) to determine target RNA levels.

The sequences of the PCR primers are as follows: Human LINC01393 forward: AGAAGTGGCTGAAACCTAAATGC; reverse: CAGGCAGAGAGAACAGCAAGT; hsa-miR-128-3p reverse transcriptional primer GTCGTATCCAGTGCAGGGTCCGAGGTATTCGCACTGGATACGACAAAGAGA; forward: GCAGTCACAGTGAACCGGT; reverse: AGTGCAGGGTCCGAGGT; NUSAP1 forward: CTGACCAAGACTCCAGCCAGAA; reverse: GAGTCTGCGTTGCCTCAGTTGT. The primers for miR-128-3p and U6 were purchased from Qiagen. The RNA level of Actin served as the internal standard. Data were analyzed by averaging triplicate Ct values.

### 3.7. Cell Transfection

Transient silencing of NUSAP1 was produced by small interfering RNAs (siRNAs) targeting NUSAP1 (5′-UUAGAGACAGGGUCUCACUGUGU-3′). The sequence of negative control siRNA (si-NC) was 5′-GAUCCGCAAAAGAGCGAAA-3′. The overexpression and inhibition of miR-128-3p were realized by miR-128-3p mimics and miR-128-3p inhibitors respectively, with NC mimics and NC inhibitors as controls (provided by QIAGEN). miR-128-3p mimic sense: 5′-TCACAGTGAACCGGTCTCTTT-3′; antisense: 5′-AAAGAGACCGGTTCACTGTGA-3′; miR-128-3p inhibitor: 5′-AAAGAGACCGGTTCACTGTGA-3′. NC sense: 5′-UUCUCCGAACGUGUCACGUUU-3′; antisense: 5′-UUAAGAGGCUUGCACAGUGCA-3′. Transfection of plasmids into GBM cells was carried out with Lipofectamine 3000 Reagent (Thermo Fisher Scientific, San Jose, CA, USA) as required.

### 3.8. LINC01393 Silencing and Overexpression

Before lentiviral transfections, the expression of LINC01393 was detected in above cell lines by qRT-PCR. The full-length of human LINC01393 cDNA was synthesized and sub-cloned into a lentiviral vector using CMV-MCS-PGK-puromycin as the main component (PHY-008, Hanyin Shanghai, China). Recombinant lentivirus and negative control lentivirus (Hanyin Shanghai, China) were prepared and titrated at 10^9^ TU (transfection units)/mL. qRT-PCR was used to confirm the overexpression/knockdown efficiency after 48 h.

A172 and U87-MG cells overexpressing LINC01393 were seeded in six-well plates (2 × 10^5^ cells/well) to establish stable cell lines. The next day, the cells were infected with virus at the same titer in the presence of 8 μg/mL Polybrene. Approximately 72 h after the virus was infected, its efficiency was evaluated under an inverted fluorescence microscope, and the medium was replaced with DMEM containing 4 g/mL puromycin. Afterward, we incubated cells for at least 14 days. The puromycin-resistant cell clones were isolated, amplified in medium containing 2 μg/mL puromycin for 7–9 days and transferred to a medium without puromycin.

### 3.9. Western Blot

After washing with ice-cold PBS three times, cells or tissues were lysed on ice in RIPA buffer (Pierce, Rockford, IL, USA) containing proteinase inhibitor (Roche, Basel, Switzerland). Protein samples (20 μg) were loaded in each lane of SDS-PAGE gels. After electrophoresis, proteins were transferred onto polyvinylidene difluoride (PVDF) membranes, which were incubated in blocking buffer (5% skim milk in TBS-T). Then, the membranes were incubated for 1 h at room temperature (RT) and then with the appropriate antibodies against NUSAP1 (Cat. No.: 12024-1-AP, Proteintech, 1:500), Phospho-NF-κB p65 (Cat. No.:3033, CST, 1:1000), NF-κB p65 (Cat. No.:10745-1-AP, Proteintech, 1:500) and β-Actin (ab6276, 1:1000, Abcam, Cambridge, MA, USA) overnight at 4 °C. After washing with TBS-T, the membranes were incubated with HRP-conjugated secondary antibodies (1:10,000 dilution) for 2 h at room temperature. Bands detections were carried out using western blot detection reagents (Odyssey).

### 3.10. Cell Counting Kit (CCK)-8 Assay

Cell viability was checked using the CCK-8 according to the manufacturer’s instructions (Dojindo, Kumamoto, Japan). All transfected cells (2 × 10^3^/100 μL cells per well) were incubated in 96-well plates for 24, 48, 72 and 96 h. The CCK-8 (10 μL) was added to each well and the plates were incubated for 2 h at 37 °C in a dark room, and then the absorbance was determined at 450-nm wavelength (OD450) using a microplate reader (BioTek Elx800; BioTek Instruments, Winooski, VT, USA).

### 3.11. Transwell Migration and Matrigel Invasion Assays

In vitro cell migration and invasion were performed using transwell chambers (Millipore, Billerica, MA, USA) with or without Matrigel. In the upper chambers, transfected glioma cells were cultured in serum-free media, while in the bottom chambers, DMEM containing 10% FBS was used. The upper chamber cells were gently removed with cotton swabs after incubation for 24 h at 37 °C. The membrane was fixed in 4% paraformaldehyde, subsequently stained with 0.3% crystal violet for 15 min and counted (at least 3 random fields of adherent cells in each well, 100× magnification).

### 3.12. Luciferase Reporter Assays

Luciferase reporter assays were carried out in HEK293 cells. The fragments of the 3’-UTR of LINC01393 or NUSAP1 containing putative miR-128-3p binding sites were amplified by PCR and subcloned into the PHY-811 vector. Next, the cells were cotransfected with reporter vector and the indicated plasmids using Lipofectamine 3000 (Life Technologies Corporation, Carlsbad, CA, USA). Assays were performed 24 h after transfection according to the Promega Dual-Luciferase Reporter Assay System protocol. The relative luciferase was calculated by normalizing firefly luciferase activity (reporter) to Renilla luciferase activity (internal control). The reporter genes containing NUSAP1-WT and NUSAP1-Mut were synthesized by Genechem (Shanghai, China).

### 3.13. RNA Immunoprecipitation Assay

The RNA immunoprecipitation (RIP) procedure was conducted using a Magna RIP Kit (Millipore, MA, USA) according to the manufacturer’s instructions. The cell lysate (100 μL) was incubated with magnetic beads conjugated with human anti-Ago2 antibody (Cat. No.: 2897, CST) or anti-IgG antibody (CST, Boston, MA, USA). Proteinase K and RNase inhibitors were then added to the samples to isolate the immunoprecipitated RNA. By using qRT-PCR, the RNA was extracted and analyzed three times.

### 3.14. Gene Set Enrichment Analysis (GSEA)

To investigate the potential molecular pathways affected by overexpression of LINC01393 and NUSAP1 in gliomas, Gene set enrichment analysis (GSEA) was hired with the hallmark gene sets and reactome signature from the Molecular Signatures Database (MsigDB, http://software.broadinstitute.org/gsea/index.jsp (accessed on 1 November 2022)) to calculate enriched pathways.

### 3.15. Animals

For the in vivo study, a total of 33 male Balb/c nude mice aged 5–6 weeks were purchased from GemPharmatech Co. Ltd. (Nanjing, China) for establishing the orthotopic intracranial glioma model. All mice were raised under specific pathogen-free condition with ambient temperature ranged from 21 to 26 °C and humidity ranged from 40% to 70%, and a light/dark cycle of 10/14-h. Food and water were available ad libitum.

### 3.16. Orthotopic Intracranial GBM Model

U87-MG cells (3 × 10^5^ cells in 5 uL of PBS) were transfected with Lenti-sh-LINC01393 or/and Lenti-sh-LINC01393-NUSAP1 overexpression (OE) and then stereotactically injected into the left striatum of mice using a small-animal stereotaxic instrument (RWD Life Science, San Diego, CA, USA) as previous reported [25]. The mice were randomly divided into three equal groups: the normal control (NC) group, the LINC01393-KD group, and the LINC01393-KD+NUSAP1-OE group. After the implantation, five mice in each group were randomly selected to detect the tumor development using magnetic resonance imaging (MRI, Bruker, BioSpec 70/30 USR, Ettlingen, Germany) at 8 and 16 days, and continued to be reared in the original environment until 50 days for survival analysis. Six mice in each group were sacrificed using cervical dislocation at 16 days, their brains and tumor tissues were harvested for hematoxylin-eosin (H&E) and qRT-PCR analysis.

### 3.17. H&E Staining

Mouse brain tissues were washed, fixed in 10% neutral-buffered formaldehyde for 24 h, dehydrated, embedded in paraffin blocks, and sectioned into 4 μm-thick slices. All slices were immersed into xylene and ethanol for dewaxing. Next, after staining with hematoxylin for 3–5 min and rinsed with tap water for 5 min, tissue sections were soaked in Hematoxylin Differentiation solution for 5 s, rinsed with tap water for another 10 min, and then immersed in Hematoxylin Scott Tap Bluing for 5 s. Following this, the sections were washed in running water for 10 min, dehydrated in graded ethanol solution (85%, 95%), stained with eosin solution for 5 min. Lastly, sections were dehydrated triple times in 100% ethanol followed by 5 min in xylene and sealed with neutral gum.

### 3.18. Statistical Analysis

In this study, the data were presented as the mean ± SD from at least 3 experimental repeats. Unpaired two-tailed Student’s t-test was used to calculate statistical significance between 2 groups. Data with comparisons to a control group over multiple timepoints were analyzed using two-way ANOVA with Dunnett’s multiple comparison test. Pearson correlation was used to evaluate the linear relationship between different gene expression levels. The Kaplan–Meier method was applied to estimate survival probability and generate survival curves for the mice. The experimental graphs were generated using GraphPad Prism 9 software. *p* < 0.05 were considered as statistically significant.

## 4. Discussion

In recent years, NUSAP1 has been reported to be a potential biomarker and therapeutic target in human cancers [11,26,27,28,29,30]. Expression of NUSAP1 in tumor cells has been linked to chemoresistance, induction of tumorigenesis, cell proliferation, migration, invasion, metastasis, and even lipid accumulation [11,21,27,28,31]. The activation of NUSAP1 is regulated by multiple upstream molecules. Guo et al. reported that miR-569 negatively regulated NUSAP1 in pancreatic cancer [21]. E2F1 might regulate NUSAP1 expression in recurrent prostate cancer through binding CCAAT box in promoter region of NUSAP1 [29]. In addition, NFYA and MYC have been implicated as transcriptional regulators of NUSAP1 [32,33]. These results suggest that transcriptional regulators of NUSAP1 can themselves be regulated at the transcriptional and posttranscriptional level. However, upstream transcriptional regulators of NUSAP1 are still unclear in GBM.

The ceRNA hypothesis is considered to be a novel post-transcriptional approach, which regulates genes by competing with miRNAs [34,35]. In the present study, according to ceRNA theory, we first predicted the potential upstream RNAs of NUSAP1 by multiple public databases and then identified lncRNA LINC01393 as a carcinogenic factor promoting glioma progression via potentially inhibit effect of miR-128-3p. Next, through both in vivo and in vitro experiments, we demonstrated that LINC01393 served as a sponge for miR-128-3p and rescued expression of NUSAP1 to promote glioma progression. This is the first study to investigate the upstream miRNA and lncRNA expression and regulation of NUSAP1 in GBM.

LINC01393 is an intergenic lncRNA located on chromosome 7q31.2, and its pathophysiological function is currently unknown. A poor prognosis of glioma was significantly correlated with overexpression of LINC01393 according to the TCGA database. Univariate and multivariate Cox regression analysis confirmed that LINC01393 was an independent prognostic factor in the TCGA LGG-GBM cohort. These results indicated that LINC01393 might be play an important role in the occurrence and development of glioma. Using FISH analysis, we noted that LINC01393 was predominantly localized in the cytoplasm of cultured GBM cells, suggesting that it may act as a ceRNA for certain miRNAs at the post-transcription level [36,37]. Overexpressed LINC01393 resulted in a significant promotion in GBM cells’ proliferation, migration, and invasion. Conversely, knockdown of LINC01393 markedly reduced these effects in vitro, inhibited the progression of the tumor in orthotopic GBM model, and extended overall survival. Results indicate that LINC01393 plays a critical role in GBM progression.

According to the miRNA-mRNA, miRNA-lncRNA negative relationship in ceRNA theory [35], we investigated the correlation among LINC01393, miR-128-3p and NUSAP1 in human GBM samples. qPCR assays showed their correlation were consistent with the previous observed results using public database. However, in Figure 3A–C, this correlation in U87-MG and U251-MG cells seems better than those in T98 and A172 cells. It could be explained that one miRNA may regulate multiple targets and one target could be co-regulated by multiple miRNAs.

Previous studies have shown that miR-128 can be used as a tumor suppressor to regulate mTOR signaling to promote apoptosis, reduce the proliferation of tumor cells and inhibit the angiogenesis and growth of gliomas by targeting mRNAs [38,39,40]. In this study, miR-128-3p has sequence complementarity to the 3′ UTR of LINC01393 and NUSAP1 mRNA. By using luciferase reporter assays, we verified the binding relationship between LINC01393 and miR-128-3p as well as miR-128-3p and NUSAP1. Afterward, RIP-qPCR assays were performed, results confirmed a significant enrichment of LINC01393, miR-128-3p, and NUSAP1 using Ago2 immunoprecipitation in U251-MG and A172 cells. Additionally, our in vitro functional experiments showed miR-128-3p mimic could rescue the inhibitory effects of LINC01393-KD, and the concomitant administration of NUSAP1-siRNA reversed this effect again. In vivo experiments showed that LINC01393-KD resulted in smaller tumor size and longer survival. qPCR analysis of mice glioma specimens confirmed that NUSAP1 expression was indeed downregulated with the decrease of LINC01393. However, upregulated NUSAP1 expression partially reversed the effect of LINC01303-KD, leading to the increased tumor volume and shorten survival. The qPCR results showed that the expression of miR-128-3p was opposite to the trend of LINC01393 and NUSAP1. Taken together, these findings supported the conclusion that LINC01393 regulated NUSAP1 expression by sponging miR-128-3p through ceRNA.

**Figure 8:**
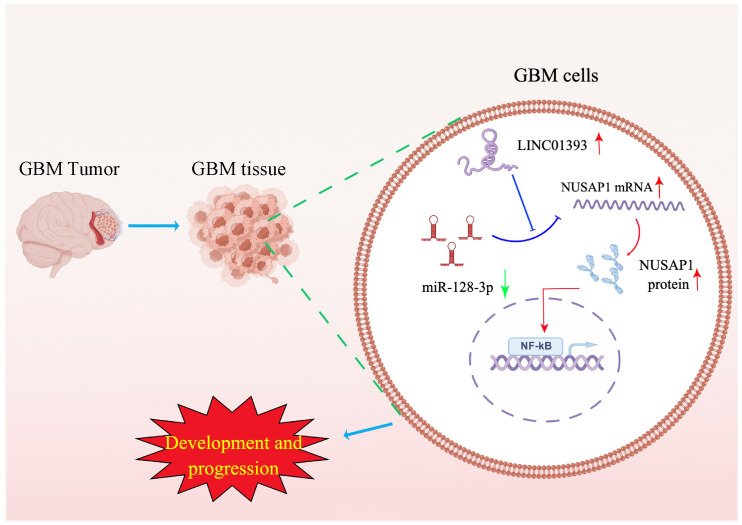
Functional mechanisms of LINC01393/miR-128-3p/NUSAP1 axis in GBM development and progression. (Red arrows show upregulation, green arrow shows downregulation, whereas T-shaped bars indicate inhibitory effects).

To explore the molecular mechanism by which LINC01393/miR-128-3p/NUSAP1 axis exerts its functions on GBM, we performed GSEA on the TCGA LGG-GBM dataset. GSEA is a bioinformatic method to evaluate whether a target gene is significantly enriched in a list of gene markers ranked by their correlation with a phenotype of interest. GSEA showed that NF-κB pathway related gene sets were positively correlated with LINC01393 and NUSAP1 expression. The NF-κB pathway is one of the most dysregulated signaling pathways in human cancers [41]. GBM cells have constitutively activated NF-κB, which promotes cell growth and survival [42]. Studies have reported that inhibiting NF-κB pathway may provide a new therapeutic target for GBM [42,43]. Therefore, we proposed that LINC01393/miR-128-3p/NUSAP1 axis may be associated with NF-κB signaling pathway in GBM. Phosphorylated p65 is critical for activation of NF-κB-dependent transcription [44]. Further immunoblotting validated that knockdown of LINC01393 decreased the phosphorylated p65 level, but overexpressed NUSAP1 could reverse this effect. This finding reflected that NF-κB signaling pathway was activated by upregulated NUSAP1. Collectively, the results showed that LINC01393/miR-128-3p/NUSAP1 axis could promote GBM development and progression via activating of NF-κB signaling pathway (Figure 8). However, how the LINC01393/miR-128-3p/NUSAP1 axis actives NF-κB pathway was not investigated in depth. Therefore, the possible regulatory mechanism of LINC01393/miR-128-3p/NUSAP1 axis still needs to be investigated in the future.

In addition to glioma, our study also found that LINC01393 showed high expression in BLCA, BRCA, CHOL, ESCA, HNSC, KICH, KIRC, LIHC, LUAD, LUSC, PRAD, and STAD, suggesting that it may play an important role in the development of these tumors (Supplementary Figure S3). Therefore, studies on the mechanism of LINC01393 in other tumors remain to be carried out. It has been reported that LncRNAs could serve as the promising biomarkers for diagnosis and therapeutics targets in various tumors. As an example, the HOXA11-AS may serve as a biomarker for identifying molecular subtypes of gliomas [45]. Prostate cancer antigen 3 (PCA3) has been approved by the FDA for use as a prostate cancer screening test [46]. Due to the fact that the number of LncRNAs is much more than that of proteins, targeted therapies against LncRNAs has received increasingly wide-ranging concerns. Using antisense oligonucleotides (ASOs) or siRNAs (also referred to as antagoNATs [47] or small activating RNAs [48]) to target long non-coding RNA can reverse the transcriptional activation effects of negative regulation. Katsushima et al. induced GSC differentiation and effectively inhibited GSC growth in an intracranial glioma xenograft mouse model with ASO intravenous treatment targeting TUG1 [49]. Özeş et al. proposed using peptide nucleic acids (PNAs) based approach to inhibit HOTAIR-EZH2 interactions and resensitize resistant ovarian tumors to platinum [50]. Combining these findings and our data presented in this study, it can be speculated that LINC01393 has great potential as a biomarker or target for anti-LncRNA therapy in the treatment of GBM.

## 5. Conclusions

In summary, the present study identifies a novel lncRNA, LINC01393, which is elevated in GBM and correlated with poor outcomes. Through a series of in vivo and in vitro experiments, our findings show that LINC01393 upregulates NUSAP1 as a ceRNA inhibiting miR-128-3p, thereby activating NF-κB pathway to promote GBM development and progression. This work reveals a novel regulatory network in GBM progression that is useful in understanding deepen mechanisms and serving as novel therapeutic targets for GBM.

## Figures and Tables

**Table 1:** Univariate and multivariate Cox regression analysis in the TCGA LGG-GBM cohort.

Characteristics	Total (N)	Univariate Analysis		Multivariate Analysis	
		Hazard Ratio (95% CI)	*p* Value	Hazard Ratio (95% CI)	*p* Value
WHO grade	614		<0.001		
G2	215	Reference		Reference	
G3	239	3.066 (2.007–4.684)	<0.001	2.323 (1.412–3.820)	<0.001
G4	160	19.142 (12.557–29.180)	<0.001	7.788 (2.476–24.501)	<0.001
IDH status	663		<0.001		
WT	237	Reference		Reference	
Mut	426	0.101 (0.076–0.134)	<0.001	0.426 (0.249–0.729)	0.002
1p/19q codeletion	666		<0.001		
Non-codel	498	Reference		Reference	
Codel	168	0.215 (0.138–0.337)	<0.001	0.765 (0.429–1.364)	0.364
Gender	672		0.123		
Female	283	Reference			
Male	389	1.219 (0.946–1.570)	0.126		
Age	672		<0.001		
≤60	533	Reference		Reference	
>60	139	4.746 (3.633–6.201)	<0.001	4.071 (2.417–6.858)	<0.001
PTO	446		<0.001		
PD	103	Reference		Reference	
SD	145	0.381 (0.250–0.580)	<0.001	0.419 (0.252–0.699)	<0.001
PR	63	0.135 (0.054–0.336)	<0.001	0.200 (0.072–0.558)	0.002
CR	135	0.129 (0.062–0.269)	<0.001	0.197 (0.092–0.423)	<0.001
LINC01393	672		<0.001		
Low	336	Reference		Reference	
High	336	2.174 (1.684–2.808)	<0.001	1.759 (1.139–2.717)	0.011

PTO: Primary therapy outcome; IDH: isocitrate dehydrogenase.

## Data Availability

All data analyzed in the present study are available in the TCGA database (http://cancergenome.nih.gov/) and Gene Expression Omnibus (GEO) database (https://www.ncbi.nlm.nih.gov/geo/) under accession number GSE4412.

## Supplementary Materials

The following supporting information can be downloaded at: https://www.mdpi.com/article/10.3390/ijms24065878/s1.

## Abbreviations

CCK-8: Cell counting kit-8; FISH: Fluorescence in situ hybridization; GBM: Glioblastoma; GSEA: Gene set enrichment analysis; LncRNA: Long non-coding RNA; NUSAP1: Nucleolar and spindle-associated protein 1; OS: overall survival; qRT-PCR: Quantitative real-time Polymerase Chain Reaction; RIP: RNA immunoprecipitation; siRNAs: Small interfering RNAs; BLCA: Bladder urothelial carcinoma; BRCA: Breast invasive carcinoma; CHOL: Cholangiocarcinoma; ESCA: Esophageal carcinoma; HNSC: Head and Neck squacmous cell carcinoma; KICH: Kidney chromophobe; KIRC: Kidney renal clear cell carcinoma; LIHC: Liver hepatocellular carcinoma; LUAD: Lung adenocarcinoma; LUSC: Lung squamous cell carcinoma; PRAD: Prostate adenocarcinoma; STAD: Stomach adenocarcinoma; GSC: Glioma stem cell.
