# Supplementary material for: LINC01393, a Novel Long Non-Coding RNA, Promotes the Cell Proliferation, Migration and Invasion through MiR-128-3p/NUSAP1 Axis in Glioblastoma
Source: Int J Mol Sci. 2023 Mar 20;24(6):5878. doi: 10.3390/ijms24065878 (PMC10056594; doi:10.3390/ijms24065878)

Figure S1. Protein coding ability prediction of LINC01393 by *InterPro*.

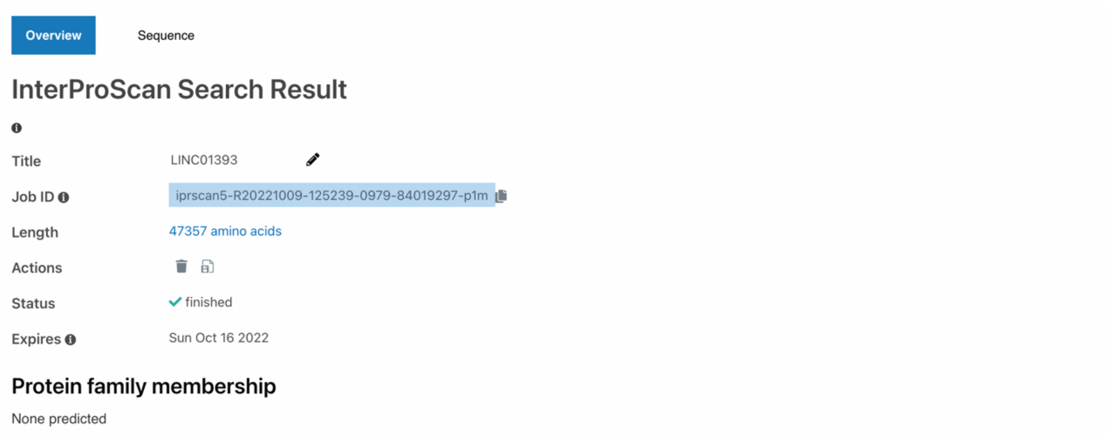

Figure S2. Representative axial T2-weighted MR images at 8 days.

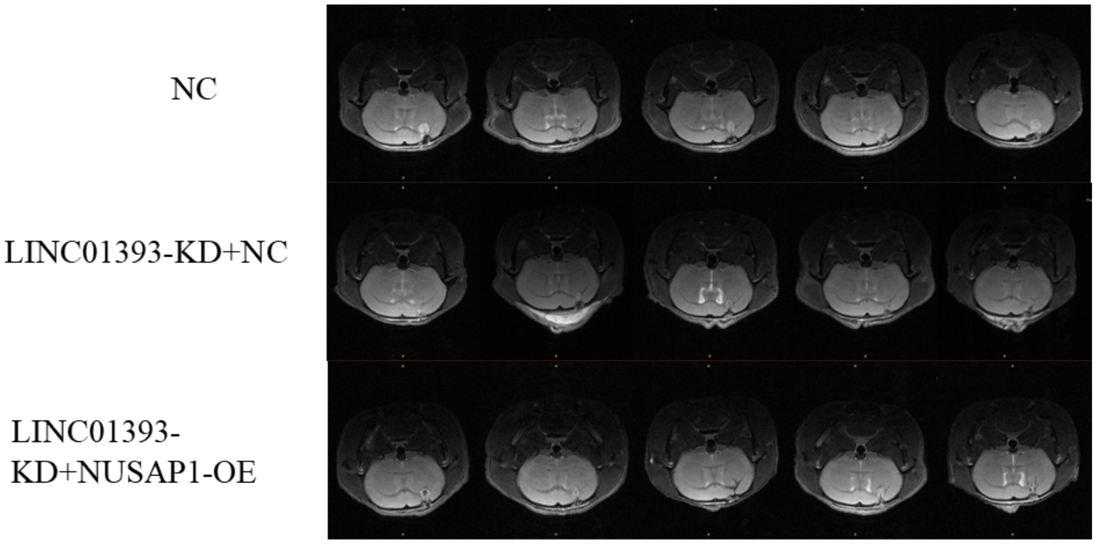

Figure S3. LINC01393 expression in pan-cancer tissues and paired non-tumor samples from TCGA database.

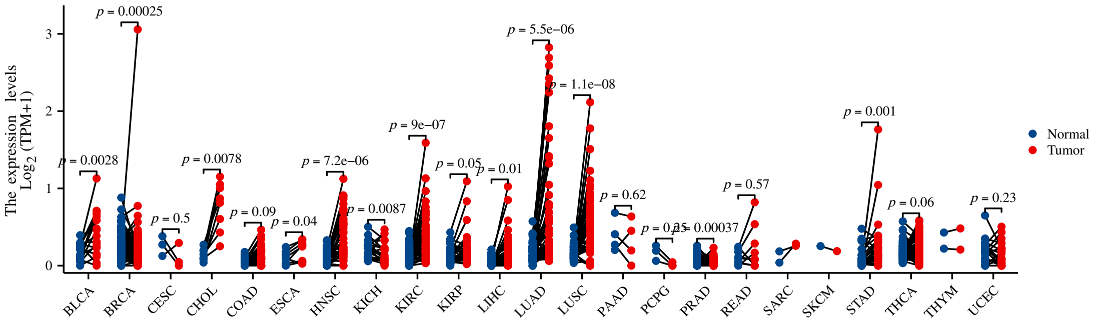

Supplement: Supplementary file 1 [file ijms-24-05878-s001.zip › Supplementary Figure S1+S2+S3.pdf]
